# Supplementary material for: Detailed molecular and epigenetic characterization of the pig IPEC-J2 and chicken SL-29 cell lines
Source: iScience. 2023 Feb 20;26(3):106252. doi: 10.1016/j.isci.2023.106252 (PMC10018572; doi:10.1016/j.isci.2023.106252)
Supplement: Data S2. Complete homer output for identified motifs in Chicken SL-29, related to Tables 5 and 6 — Homer motif analysis results for histone modifications H3K4me1, H3K4me3, H3K27ac, enhancers, and ATAC-seq of chicken SL-29 cell line. Parameters for possible false positives is as mentioned earlier for S5. [file mmc3.zip › Data_S2/S6/Chicken_SL_29/peak_files_H3K27ac/homerResults/motif13.similar.html]

motif13

## Information for motif13

C
A
G
T
C
A
G
T
A
G
C
T
T
A
G
C
C
G
A
T
A
T
C
G
A
T
G
C
A
T
G
C
  
Reverse Opposite:  

T
A
C
G
T
A
C
G
T
A
G
C
G
C
T
A
A
T
C
G
C
T
G
A
G
T
C
A
G
T
C
A
  

|  |  |
| --- | --- |
| p-value: | 1e-33 |
| log p-value: | -7.714e+01 |
| Information Content per bp: | 1.597 |
| Number of Target Sequences with motif | 14214.0 |
| Percentage of Target Sequences with motif | 47.13% |
| Number of Background Sequences with motif | 13193.3 |
| Percentage of Background Sequences with motif | 43.66% |
| Average Position of motif in Targets | 149.7 +/- 84.7bp |
| Average Position of motif in Background | 148.9 +/- 89.8bp |
| Strand Bias (log2 ratio + to - strand density) | 0.0 |
| Multiplicity (# of sites on avg that occur together) | 1.38 |
| Motif File: | file (matrix) reverse opposite |

### Similar de novo motifs found

|  |  |  |  |  |  |  |  |
| --- | --- | --- | --- | --- | --- | --- | --- |
| Rank | Match Score | Redundant Motif | P-value | log P-value | % of Targets | % of Background | Motif file |
| 1 | 0.608 | A T C G A G C T A C T G A C G T A G C T A T G C C T A G A C T G A G T C A T G C | 1e-22 | -52.100678 | 0.58% | 0.25% | motif file (matrix) |
| 2 | 0.616 | A G T C A C G T A C G T G T A C A G T C A C T G G T A C A G T C | 1e-17 | -40.995159 | 1.84% | 1.25% | motif file (matrix) |
| 3 | 0.682 | A C T G A C T G C G T A A C T G C G T A C G T A C G T A A G T C A C T G A G T C | 1e-8 | -18.911294 | 0.12% | 0.04% | motif file (matrix) |
